# A Multicenter Before-After Study on Reducing Unnecessary Diagnostics by Changing the Attitude of Caregivers: Protocol for the RODEO Project

**DOI:** 10.2196/10473

**Published:** 2018-08-21

**Authors:** Renuka S Bindraban, Marlou LH van Beneden, Mark HH Kramer, Wouter W van Solinge, Suzanne IM Neppelenbroek, Merel van Wijnen, Anita Griffioen-Keijzer, Muhammad Al-Dulaimy, Maarten J ten Berg, Prabath WB Nanayakkara

**Affiliations:** ^1^ Acute Medicine Section Department of Internal Medicine VU University Medical Center Amsterdam Netherlands; ^2^ Department of Clinical Chemistry and Hematology University Medical Center Utrecht Utrecht Netherlands; ^3^ Department of Internal Medicine North-West Hospital Group Alkmaar Alkmaar Netherlands; ^4^ Department of Clinical Chemistry Meander Medical Center Amersfoort Netherlands; ^5^ Department of Internal Medicine Spaarne Gasthuis Haarlem Netherlands; ^6^ Department of Internal Medicine Spaarne Gasthuis Hoofddorp Netherlands; ^7^ Department of Internal Medicine Zaans Medical Center Zaandam Netherlands

**Keywords:** diagnostic laboratory test, diagnostic testing, protocol, implementation

## Abstract

**Background:**

Appropriate use of diagnostic laboratory tests is challenging, and estimates of 20% for overutilization and 45% for underutilization have been reported. Introducing effective and sustainable solutions to stimulate optimal use of laboratory testing in clinical practice is a challenge. A recent pilot study from our group, focusing on increasing the awareness about appropriate laboratory testing with the aim of changing the mindset of health care workers, has shown promising results. In this project, we aim to extend this multistep intervention to the internal medicine departments of 4 large Dutch hospitals. We aim to reduce unnecessary laboratory testing by 5%.

**Objective:**

Our primary objective is to determine the effect of our intervention on diagnostic laboratory test order volume. Our secondary objectives are to determine the effect of our intervention on laboratory expenditure and order volumes, expenditures for other diagnostic modalities, and clinical patient outcomes. We will also analyze the barriers and facilitators for deimplementation of unnecessary laboratory testing.

**Methods:**

The main interventions of this before-after study will be an intensified supervision of residents by experienced physicians regarding test ordering, creating awareness through education and monthly feedback on ordering patterns, and changes in (computerized) order entry systems.

**Results:**

At the time of publication of this protocol, the project is in the phase of data collection. We expect to present data on reduction early in the fourth quarter of 2018.

**Conclusions:**

In this project, we aim to reduce the unnecessary diagnostic testing in the internal medicine departments of 4 teaching hospitals. Although the main interventions will be similar, each clinic is given the opportunity to focus on the specific facets of the interventions as deemed useful according to the local situation. If effective, the study provides a framework for a nationwide initiative for reducing inappropriate laboratory testing.

**Registered Report Identifier:**

RR1-10.2106/10473

## Introduction

Over the past decades, a marked rise in health care expenses has been observed in Western countries. In the Netherlands, the burden of health care on the gross domestic product has increased from 7.9% in 1998 to 10.5% in 2016, corresponding to an increase from approximately 30.9 to 73.7 billion euros. A large part of the total health care expenditure consists of hospital care, including diagnostic testing [1,2]. The volume, and consequently the costs, of performing diagnostic tests is increasing, with earlier studies reporting a doubling of the rate every 5-10 years over the past decades [3].

In 2015, Kobewka et al [4] reviewed numerous international studies and concluded that a considerable proportion of performed (laboratory) tests were unnecessary, that is, they did not contribute to patient care. A review addressing the appropriateness of diagnostic laboratory testing, as judged by the presence of multiple appropriateness criteria (eg, criteria based on testing frequency, choice of test compared with possible alternatives, and probability of abnormal test results), has reported a mean rate of overutilization of approximately one-fifth from 1997 to 2012 [5]. Consequently, laboratory testing is often targeted in efforts to reduce health care expenditure. Besides the financial impact, overutilization increases the number of false-positive results, which leads to more, sometimes invasive and potentially harmful, tests [6]. Also, excessive blood draw can result in iatrogenic anemia and can lead to less patient-friendly practice, for example, through painful punctures and unnecessary trips to the hospital [7].

In 2009, a multifaceted intervention focusing mainly on laboratory test reduction was implemented at the internal medicine department of the VU University Medical Center (VUmc). Utilization of other diagnostics, such as radiology, declined too. Our efforts resulted in a 13% gross reduction in diagnostic expenditure compared with that in the previous year. When extrapolating these results, nationwide implementation of these interventions could result in a potential saving of millions of euros.

In the “Reduction of unnecessary diagnostics through attitude change of the caregivers” (RODEO) project, we will assess the effects of a multifaceted intervention aimed at improving awareness about (in)appropriate laboratory testing on the volume and costs of diagnostic testing and clinical outcomes of patients in the internal medicine departments of multiple peripheral teaching hospitals over 6 months. We aim to reduce (unnecessary) diagnostic testing by 5%. Our primary focus will be on laboratory testing, although we will also assess the effects of our intervention on the volume and costs of other diagnostics modalities. In addition, we will assess the sustainability of the interventions during an additional 8-month period. We will also analyze the process of deimplementation of unnecessary laboratory testing in the participating hospitals, aiming to identify barriers and facilitators.

This project is a part of the “To do or not to do? Reducing low-value care” program aimed at reducing low-value care [8]. The program was initiated by the Dutch Federation of University Medical Centers.

## Methods

### Study Design and Setting

This multicenter before-after study was conducted at the internal medicine departments (inpatient, outpatient, and emergency departments) of the Zaans Medical Center (Zaandam), Meander Medical Center (Amersfoort), North-West Hospital group (location Alkmaar), and Spaarne Gasthuis (locations Haarlem and Hoofddorp), which are all teaching hospitals in the Netherlands; in the rest of the document, we have referred to these participating hospitals anonymously as hospital 1-4.

Access to timely data on volume and costs of different diagnostic modalities (laboratory, radiology, microbiology, pathology, and nuclear medicine) for the duration of the project and for the 3 preceding years was a criterion for inclusion. Another criterion for inclusion was consent of the participating hospital’s Board of Directors. The project protocol was assessed by the Medical Ethics Review Committee of VUmc. They determined that the Medical Research Involving Human Subjects Act does not apply to this project and that official approval by the Medical Ethics Review Committee is not required. Local feasibility was approved by the local ethics committees and Board of Directors of all participating hospitals. Data were collected anonymously.

### Deimplementation Strategy

The study consists of 3 time periods: 3-4 months of preintervention, 6 months of intervention, and 8 months of postintervention. The study was started in August 2016; after the study period ends, we plan to continue monitoring these interventions to assess sustainability.

Before the start of the preintervention period, the internal medicine departments of the participating hospitals were contacted and informed about the project. Upon inclusion of a department, cooperation agreements were signed by the principle investigator of the hospital, and thereafter, a project team consisting of a senior internist (ambassador), internal medicine resident, a business intelligence collaborator, and a clinical chemist were formed.

### Preintervention Period (3-4 Months)

During the preintervention period, data on volume and costs of diagnostics as well as on patient outcomes from the previous 3 years were collected. Also, data on the characteristics of the participating departments such as the number and years of experience of residents and supervising physicians, methods and frequency of supervision of residents, and characteristics of ordering systems were collected. The preintervention period started in August 2016 at hospitals 1 and 2, in September 2016 at hospital 3, and in November 2016 at hospital 4.

### Intervention Period (6 Months)

At the start of the intervention period, a launching conference took place with the members of all the participating project teams. Each project team was requested to give a presentation on the characteristics of their department, data on their ordering patterns over the previous years, and previous projects related to this topic. In addition, each project team was requested to present interventions tailor-made for their department structure.

We also assessed foreseen barriers and facilitators for deimplementation and discussed how to tackle them, if necessary. The program of this launching conference can be found in Multimedia Appendix 1.

Upon starting the intervention period, data collected in the preintervention period and planned interventions were presented by the local project teams to the caregivers working in their departments. During the intervention period, the local project teams performed the interventions and had frequent periodic progress meetings with the coordinating project team. The interventions performed and how they were developed have been described in more detail in the subsection “Description of interventions.”

A second conference was organized in which the project teams presented their results from the initial months, exchanged experiences and ideas on how to proceed in the remaining months of the project, and discussed how to sustain the effects after the termination of the active intervention period. The program of this conference can be found in Multimedia Appendix 1.

The intervention period started in November 2016 at hospitals 1 and 2, in January 2017 at hospital 3, and in March 2017 at hospital 4.

### Postintervention Period (8 Months)

In the postintervention period, the sustainability of the intervention was analyzed. During this period, a third joint conference was organized with all the participating project teams in which the project teams were requested to present their results and exchange experiences and ideas on how to further sustain the achieved effects. The program of this conference can be found in Multimedia Appendix 1. Data on diagnostic volume and costs and patient outcomes were reanalyzed.

The postintervention period started in May 2017 at hospitals 1 and 2, in July 2017 at hospital 3, and in September 2017 at hospital 4. The postintervention period ended in December 2017 at hospitals 1 and 2, in February 2018 at hospital 3, and in April 2018 at hospital 4. We will continue to monitor the progress and results for 12 months.

At the time of publication of this protocol, the project is in the data collection phase.

### Description of Interventions

Target items for interventions were determined by the project team from different angles: tests that are known to be frequently overused, tests ordered in high frequency or generating high costs to the department, and diagnosis-related groups occurring in high frequency or generating high costs (compared with the benchmark, when available). All participating hospitals were given the opportunity to focus on the specific facets of the intervention as deemed useful in the local situation, thus, “tailoring” their interventions.

The interventions performed in this project were partly derived from previous literature [4,9], in which the interventions were divided into the following categories: education, audit and feedback methods, (computerized) provider order entry system changes, and others. To develop and classify the interventions in the RODEO project, we used slightly different categories.

The main interventions were intensified supervision, creating awareness, and modifications in (computerized) order entry systems. Intensified supervision of residents by senior physicians refers to explicitly focusing on indications for ordering laboratory tests and asking critical questions (“Does the result of this test add value for diagnostics, treatment, or prognosis?” “Is repetition of this test necessary at this moment?” “Is it necessary to order these tests combined?”) during morning reports, daily supervision meetings, grand rounds, and other clinical meetings.

In addition to paying more attention to laboratory ordering, awareness was also created through educational sessions or emails, posters displaying recommendations and general agreements on ordering of (specific) tests, and distribution of pocket-cards containing charges for frequently ordered tests. Awareness was also created by providing feedback on (changes in) ordering patterns to the physicians working in the department.

Modifications in (computerized) order entry systems included instating time limits on ordering tests for which a repeat test is not necessary within a certain time interval and modification of existing order panels.

The coordinating project team and the local project teams held monthly meetings during the intervention period and bi- or tri-monthly meetings during the postintervention period. In these meetings, the progress of (development of) each intervention was discussed. Also, changes in total order volume and costs were discussed using data acquired from Business Intelligence or Business Control collaborator. If explicit focus was placed on specific tests, changes in the order volume of these tests were discussed separately.

**Figure 1:**
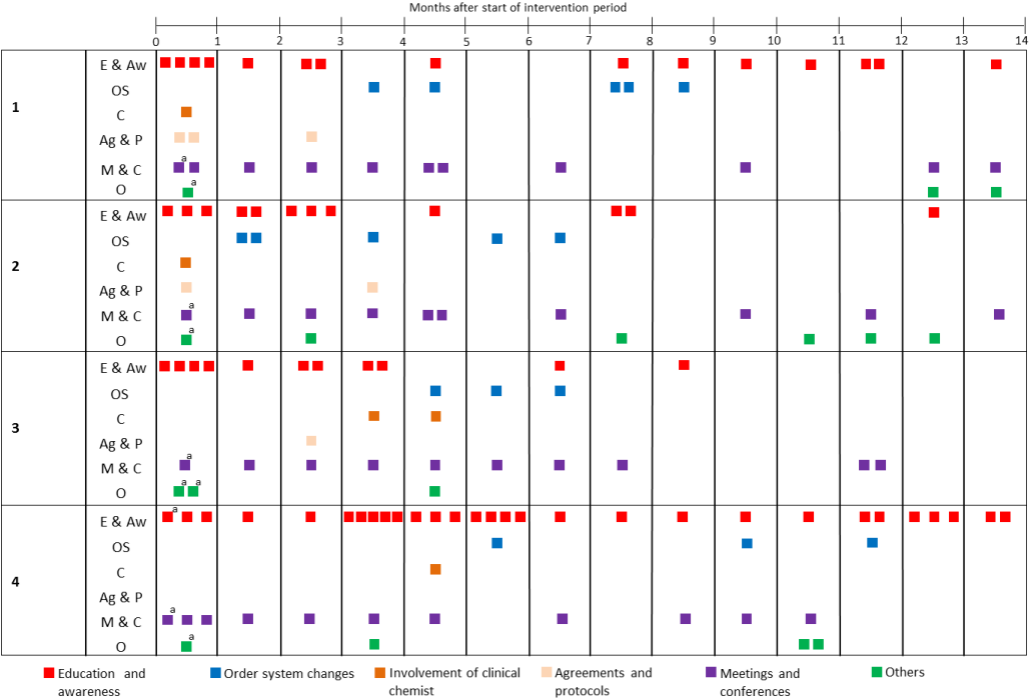
Timeline of interventions. a: action took place before the intervention period. E & Aw: education and awareness; OS: order system changes; C: involvement of clinical chemist; Ag & P: agreements and protocols; M & C: meetings and conferences; and O: others.

The interventions performed in each clinic, classified by category, are displayed in Figure 1. Details on each intervention can be found in Multimedia Appendix 2.

### Endpoints and Data Collection

In the RODEO project, we aim to reduce the amount of (unnecessary) diagnostic laboratory testing. Based on previous experience from our pilot study, we decided to aim for a conservative estimate of 5% reduction in total test volume.

### Primary Endpoint

The primary endpoint is diagnostic laboratory test order volume in the internal medicine department (inpatient, outpatient, and emergency).

Laboratory test order volume will be assessed as the total number of orders for laboratory tests and will be corrected for patient census using “standardized patient units,” a measure that will be calculated using the numbers of admissions, in-hospital admission days, day care admissions, and number of first outpatient consultations [10]. Order volume and data required for calculation of the number of standardized patient units will be acquired through the Department of Business Intelligence or Business Control and the Department of Clinical Chemistry.

### Secondary Endpoints

Secondary endpoints are laboratory expenditure, order volumes and expenditure for other diagnostic modalities, and clinical patient outcomes.

Laboratory expenditure will be assessed as total expenditure and corrected for patient census. Order volumes and expenditure (if possible) for other diagnostic modalities (radiology, microbiology, pathology, and nuclear medicine considered separately) will be assessed as the total number or costs of orders and will also be corrected for patient census.

To ensure that a reduction in diagnostic testing does not affect patient outcomes, we will take into account clinical patient outcomes before and after the intervention based on duration of hospital stay, 30-day readmission rate, rate of repeated outpatient visits relative to first outpatient visits, and glycated hemoglobin.

Expenditure, order volumes, data required for calculation of the number of standardized patient units, and data on clinical outcomes will be acquired through the Department of Business Intelligence or Business Control and the Department of Clinical Chemistry.

### Evaluation of Barriers and Facilitators

An important part of the RODEO project is evaluating the barriers and facilitators of deimplementation of unnecessary laboratory testing. To identify these factors, questionnaires (Multimedia Appendix 3) on these topics were administered to each project team during the (pre)intervention period. During the remainder of the project, these factors were discussed during multiple conferences.

### Statistical Analysis

All statistical analyses will be performed using R version 3.4.2. We will assess the volume of diagnostic tests ordered (total volume and volume of laboratory, radiology, microbiology, pathology, and nuclear medicine tests separately) during the year after the start of the intervention (ie, intervention period and postintervention period) and the preceding years.

We will adjust for patient census using “standardized patient units,” a concept previously used by Dutch insurance companies for reimbursement purposes. The number of standardized patient units will be calculated using the following formula:

(10 × number of admissions) + (0.5 × number of patient days) + (3.5 × number of day admissions) + (1.2 × number of first outpatient consultations)

An interrupted time series analysis will be performed to assess the effects of the intervention on test volume. We will use an autoregressive integrated moving average model to analyze whether the intervention led to a (more profound) change in the number of tests per standardized patient unit after the intervention. We will adjust for seasonal variation.

## Results

We expected the study period to end in April 2018. Furthermore, we expect to be able to present data on reduction early in the fourth quarter of 2018.

## Discussion

In this protocol, we have described the objective, design, deimplementation strategy, and endpoints of the RODEO project, aiming to reduce unnecessary diagnostic testing in the internal medicine departments of 4 large teaching hospitals in the Netherlands.

The approach used in this project was derived from an approach previously used in a pilot project within different departments of VUmc [1]. In this project, a senior physician was designated as “ambassador” or “local champion” who was responsible for coordinating and performing the interventions in the participating departments, which consisted mainly of intensified supervision, education, and feedback. During this pilot project, no modifications were made in the (computerized) order entry system. Although commitment of a supervisor has been shown to play a crucial role in the success of a project, the VUmc project identified a prominent role for residents as one of the key success factors. Furthermore, the VUmc study team found that the Clinical Chemistry department played an important role in the pilot project. Therefore, we appointed a central project team at each participating department consisting of an internal medicine supervisor and a resident, a clinical chemist, and a collaborator from the Department of Business Intelligence or Business Control.

Although the main interventions were intensified supervision, creating awareness through education and feedback, and changes in (computerized) order entry systems, each hospital was given the opportunity to focus on the specific facets of the interventions as deemed useful in the local situation. Each clinic, thus, had the opportunity to “tailor” its interventions as deemed fit, which can be considered as a strength of our approach. Another strength of our project is the inclusion of 4 relatively large teaching hospitals. A potential limitation of our approach is the nonexistence of a control group. Also, it was not possible to determine the effect of individual aspects of this multistep intervention due to the limited time available for this project. Furthermore, we did not include patients in our efforts to reduce laboratory testing. We expect the study period to end in April 2018. If effective, this study will provide a framework for a nationwide initiative for reducing inappropriate laboratory testing.

## Appendix

Multimedia Appendix 1 Program conferences.

Multimedia Appendix 2 Interventions by hospital.

Multimedia Appendix 3 Questionnaire "Willingness to Change".

## Abbreviations

RODEO: Reduction of unnecessary diagnostics through attitude change of the caregivers
