# Supplementary material for: A Multicenter Before-After Study on Reducing Unnecessary Diagnostics by Changing the Attitude of Caregivers: Protocol for the RODEO Project
Source: JMIR Res Protoc. 2018 Aug 21;7(8):e10473. doi: 10.2196/10473 (PMC6123537; doi:10.2196/10473)
Supplement: Multimedia Appendix 1 [file resprot_v7i8e10473_app1.pdf]

Program launching conference RODEO project
Thursday September 29th, 2016
Location: Olympic Stadium Amsterdam

12.00-13.00	Lunch
13.00-13.30	Introduction by coordinating project team
13.30-14.15	Introductory presentation by participating project teams (5 – 6 minutes per project team)
14.15-14.45	Brainstorm by project team members within their own discipline: assessment of foreseen barriers and facilitators
14.45-15.15	Developing a plan for reduction by each project team
15.15-15.45	*Coffee break*
15.45-16.45	Presentation of developed plan for reduction by each project team (8 minutes per project team)
16.45-18.00	Discussion

Program second conference RODEO project
Wednesday March 22th, 2017
Location: VU University Medical Center

17.30-18.00	*Dinner*
18.00-18.10	Introduction
18.10-18.50	Presentation of current situation by participating project teams (10 minutes per project team)
18.50-19.00	Presentation on national initiative aiming to integrate cost-consciousness into resident training programs ("Bewustzijnsproject")
19.00-19.10	*Coffee break*
19.10-19.40	Brainstorm by project team members within their own discipline:<br>• Do's and Don'ts<br>• Points of concern for sustainability
19.45-20.00	Follow-up agreements
20.00	Discussion

Program third conference RODEO project

Wednesday December 13th, 2017
Location: VU University Medical Center

17.30-18.00	*Dinner*
18.00-18.10	Introduction
18.10-19.10	Presentation of current situation by participating project teams (10 minutes per project team)
19.10-19.30	*Coffee break*
19.30-20.30	Learned lessons: what works, what doesn’t work?<br>• Education<br>• Time limits, protocols and agreements<br>• Preliminary conclusions
20.30-20.45	Publication of results and experiences<br>Discussion
