# Supplementary material for: A Multicenter Before-After Study on Reducing Unnecessary Diagnostics by Changing the Attitude of Caregivers: Protocol for the RODEO Project
Source: JMIR Res Protoc. 2018 Aug 21;7(8):e10473. doi: 10.2196/10473 (PMC6123537; doi:10.2196/10473)
Supplement: Multimedia Appendix 2 [file resprot_v7i8e10473_app2.pdf]

Interventions–Hospital 1

Education and awareness
November 2016:
- Presentation: Introduction of project and diagnostic test ordering patterns (volume and costs)
- Educational session: Antinuclear Antibody (ANA) testing
- Brainstorm meeting on possible targets for interventions
- Distribution of mouse pad with questions to keep in mind when ordering laboratory tests: “Does the result of this test have added value for diagnostics, treatment of prognosis?” “Is repetition of this test necessary at this moment?” “Is it necessary to order these tests combined?”

December 2016:
- Educational session: Amylase and lipase

January 2017:
- Educational session: Blood cultures
- Presentation: Feedback on diagnostic test ordering patterns (volume, costs, number of phlebotomies)

March 2017:
- Distribution of pocket-cards containing charges for commonly used tests

June 2017:
- Educational session: Liver biochemistry

July 2017:
- Educational session: Kidney function panel

August 2017:
- Educational session: Fecal cultures

September 2017:
- Presentation: Feedback on diagnostic test ordering patterns (volume, costs, number of phlebotomies) to Internal Medicine staff

October 2017:
- Educational session: Analysis of anemia
- Presentation: Principles of project to residents and interns from all departments

December 2017:
- Educational session: Cardiac enzymes in patients with chest pain at the inpatient department

Order system changes
February 2017:
- Modification of “Geriatrics” order panel

March 2017:
- Instatement of time limits on test orders: repetitive order within prespecified time frame is automatically rejected, can only be overruled after direct contact with the laboratory

June 2017:
- Abolishment of amylase testing by the laboratory
- Modification to order system ensuring fast display of performed blood culture tests while awaiting result

July 2017:
- Introduction of “kidney function” panels WITH or WITHOUT urea

Clinical chemist
November 2016:
- Introduction of presence and participation in 1-2 grand rounds a week by clinical chemist.

Agreements and protocols

November 2016:

- Introduction of general agreement: Limit the frequency of laboratory testing to two times a week in clinically stable patients
- Introduction of general agreement: Limit the frequency of CRP testing to three times a week

January 2017:

- Introduction of general agreement: Instruction to care providers to clearly indicate which tests are to be requested by medical secretaries (eg, list tests to be requested individually instead of ordering “kidney panel”)

Meetings and conferences

September 2016:

- Launching conference with project teams of all participating departments and coordinating project team

November 2016:

- Monthly progress meeting (1) project team and coordinating project team

December 2016:

- Monthly progress meeting (2) project team and coordinating project team

January 2017:

- Monthly progress meeting (3) project team and coordinating project team

February 2017:

- Monthly progress meeting (4) project team and coordinating project team

March 2017:

- Monthly progress meeting (5) project team and coordinating project team
- Conference with project teams of all participating departments and coordinating project team

May 2017:

- Monthly progress meeting (6) project team and coordinating project team

August 2017:

- Progress meeting (7) project team and coordinating project team

November 2017:

- Progress meeting (8) project team and coordinating project team

December 2018:

- Conference with project teams of all participating departments and coordinating project team

Others

October 2016:

- Presentation: Introduction of project to Board of Directors

November 2017:

- Display of posters in workspaces stating important RODEO principles

December 2017:

- Introduction of e-mail to inform new employees to the RODEO project and its main principles

Interventions–Hospital 2

Education and awareness
November 2016:
- Presentation: Introduction of project and diagnostic test ordering patterns (volume and costs)
- Distribution of pocket-cards containing charges for commonly used tests
- Distribution of mouse pad with questions to keep in mind when ordering laboratory tests: “Does the result of this test have added value for diagnostics, treatment of prognosis?” “Is repetition of this test necessary at this moment?” “Is it necessary to order these tests combined?”

December 2016:
- Educational session: Local guideline on “Chronic fatigue”
- Newsletter: Frequency of CRP testing, combining liver panel tests

January 2017:
- Newsletter: Sodium testing
- Educational session: Amylase and lipase
- Educational session: Value of routine hemoglobin measurement after kidney biopsy

March 2017:
- Educational session: “Standard Internal Medicine” order panel

June 2017:
- Presentation: Principles of project at hospital-wide meeting
- Presentation: Principles of project to residents and interns from all departments

November 2017:
- Presentation: Feedback on diagnostic test ordering patterns (volumes and costs) to Internal Medicine staff

Order system changes
December 2016:
- Sequential ordering, pop-up upon ordering TSH: FT4 value will automatically be determined if TSH value deviates from normal. This pop-up was deleted in February 2017 due to provider complaints.
- Sequential ordering: Anti-IA2 will only be performed when anti-GAD is negative

February 2017:
- Instatement of time limits on test orders: repetitive order within prespecified time frame is automatically rejected, can only be overruled after direct contact with the laboratory

April 2017:
- Modification to order system ensuring fast display of performed blood culture tests while awaiting result

May 2017:
- Modification of ‘Emergency Department’ order panel

Clinical chemist
November 2016:
- Introduction of presence and participation in 1-2 grand rounds a week by clinical chemist

Agreements and protocols
November 2016:
- Introduction of general agreement: Limit the frequency of laboratory testing to two times a week in clinically stable patients

February 2017:

- Modification of local guideline for laboratory diagnostics in hemodialysis patients

Meetings and conferences
September 2016:
- Launching conference with project teams of all participating departments and coordinating project team

December 2016:
- Monthly progress meeting (1) project team and coordinating project team

January 2017:
- Monthly progress meeting (2) project team and coordinating project team

February 2017:
- Monthly progress meeting (3) project team and coordinating project team

March 2017:
- Monthly progress meeting (4) project team and coordinating project team
- Conference with project teams of all participating departments and coordinating project team

May 2017:
- Monthly progress meeting (5) project team and coordinating project team

August 2018:
- Progress meeting (6) project team and coordinating project team

October 2017:
- Progress meeting (7) project team and coordinating project team

December 2018:
- Conference with project teams of all participating departments and coordinating project team

Others
October 2016:
- Presentation: Introduction of project to Board of Directors

January 2017:
- Involvement of specialists representing Internal Medicine sub-specialisms (Nephrology, Oncology) within project team

June 2017:
- Presentation: Feedback on diagnostic test ordering patterns (volumes and costs) to Board of Directors

September 2017:
- Involvement of second resident within project team

October 2017:
- Display of posters in workspaces stating important RODEO principles

November 2017:
- Presentation: Feedback on diagnostic test ordering patterns (volumes and costs) to Board of Directors

Interventions–Hospital 3

Education and awareness

January 2017:

- Presentation: Introduction of project and diagnostic test ordering patterns (volume and costs)
- Educational session: Blood cultures
- Newsletter: Costs for laboratory diagnostics at hospital 3, Amylase and lipase
- Distribution of mouse pad with questions to keep in mind when ordering laboratory tests: “Does the result of this test have added value for diagnostics, treatment of prognosis?” “Is repetition of this test necessary at this moment?” “Is it necessary to order these tests combined?”

February 2017:

- Newsletter: Arterial versus venous blood gas analysis, D-dimer, Choosing Wisely campaign, Urine testing

March 2017:

- Newsletter: Creatinine and urea, Costs for diagnostic testing at hospital 3
- Newsletter: ASAT and ALAT, Importance of input from specialists for RODEO

April 2017:

- Presentation: Feedback on diagnostic test ordering patterns (volumes and costs)
- Newsletter: Volume and costs of diagnostic testing at hospital 3, Abdominal X-ray, Imaging for diverticulitis

July 2017:

- Newsletter: Charges per laboratory order, Volume and costs of 25 most frequently requested laboratory tests

September 2017:

- Presentation: Feedback on diagnostic test ordering patterns (volumes and costs)

Order system changes

May 2017:

- Modification “Pulmonary Medicine” and “Internal Medicine” ordersets

June 2017:

- Instatement of time limits on test orders: repetitive order within prespecified time frame is automatically rejected, can only be overruled after direct contact with the laboratory

July 2017:

- Adjustment of previously instated time limits due to complaints

Clinical chemist

April 2017:

- Introduction of presence and participation in 1-2 grand rounds a week by clinical chemist

May 2017:

- Involvement of second clinical chemist within project team

Agreements and protocols

March 2017:

- Introduction of working agreement: Tests to be performed after patient discharge are to be requested by treating physician instead of by medical secretaries

Meetings and conferences

September 2016:

- Launching conference with project teams of all participating departments and coordinating project team

February 2017:
- Monthly progress meeting (1) project team and coordinating project team

March 2017:
- Conference with project teams of all participating departments and coordinating project team

April 2017:
- Monthly progress meeting (2) project team and coordinating project team

May 2017:
- Monthly progress meeting (3) project team and coordinating project team

June 2017:
- Monthly progress meeting (4) project team and coordinating project team

July 2017:
- Monthly progress meeting (5) project team and coordinating project team

August 2017:
- Progress meeting (6) project team and coordinating project team

December 2017:
- Progress meeting (7) project team and coordinating project team
- Conference with project teams of all participating departments and coordinating project team

<u>Others</u>

November 2016:
- Presentation: Introduction of project to Board of Directors
- Presentation: Introduction of project to Internal Medicine specialists within department

May 2017:
- Involvement of specialists representing Internal Medicine sub-specialisms (Nephrology, Oncology, Gastro-enterology), no active role in project team

Interventions–Hospital 4

<u>Education and awareness</u>

September 2016:

- Presentation: Introduction of project

March 2017:

- Distribution of pocket-cards containing charges for commonly used tests
- Distribution of mouse pad with questions to keep in mind when ordering laboratory tests: "Does the result of this test have added value for diagnostics, treatment of prognosis?" "Is repetition of this test necessary at this moment?" "Is it necessary to order these tests combined?"

April 2017:

- Educational session: Health care costs in the Netherlands, ASAT testing

May 2017:

- Educational session: Urea

June 2017:

- Educational session: Arterial blood gas analysis
- Educational session: ASAT
- Educational session: Blood cultures
- Educational session: Amylase and lipase
- Newsletter: Information on RODEO project in department newsletter

July 2017:

- Educational session: Iron, ferritin, transferrin
- Educational session: Abdominal X-ray
- Educational session: Vitamins

August 2017:

- Educational session: Urine testing
- Educational session: CRP
- Educational session: Blood products
- Educational session: MLPA

September 2017:

- Educational session: (NT-pro)BNP

October 2017:

- Educational session: Actual deviation or natural fluctuation?

November 2017:

- Educational session: Rheumatoid factor and anti-CCP

December 2017:

- Educational session: Urine antigen test

January 2018:

- Educational session: Erythrocyte Sedimentation Rate

February 2018:

- Educational session: Previously discussed subject
- Educational session: Previously discussed subject

March 2018:

- Educational session: Previously discussed subject
- Educational session: Previously discussed subject
- Educational session with ED personnel: Indications for arterial blood gas analysis

April 2018:

- Educational session: Troponin
- Educational session: Previously discussed subject

Order system changes

August 2017:

- Removal of order panels that are infrequently used: “Deep Venous Thrombosis” and “Sepsis”

December 2017:

- Modification of “Hematologic IC” order panel

February 2018:

- Instatement of time limits on test orders: repetitive order within prespecified time frame triggers an alert on redundancy

Clinical chemist

July 2017:

- Introduction of presence and participation in 1 grand round a week by clinical chemist

Meetings and conferences

September 2016:

- Launching conference with project teams of all participating departments and coordinating project team

March 2017:

- Monthly progress meeting (1) project team and coordinating project team
- Conference with project teams of all participating departments and coordinating project team

April 2017:

- Monthly progress meeting (2) project team and coordinating project team

May 2017:

- Monthly progress meeting (3) project team and coordinating project team

June 2017:

- Monthly progress meeting (4) project team and coordinating project team

July 2017:

- Monthly progress meeting (5) project team and coordinating project team

September 2017:

- Progress meeting (6) project team and coordinating project team

November 2017:

- Progress meeting (7) project team and coordinating project team

December 2017:

- Conference with project teams of all participating departments and coordinating project team

January 2018:

- Progress meeting (8) project team and coordinating project team

Others

September 2016:

- Presentation: Introduction of project to Board of Directors

June 2017:

- Involvement of Nephrologist and Nephrology resident within project team

January 2018:

- Addition of the RODEO project and its main principles as topic in introductory meetings for new employees
- Display of posters in workspaces stating important RODEO principles
