# Supplementary material for: A Multicenter Before-After Study on Reducing Unnecessary Diagnostics by Changing the Attitude of Caregivers: Protocol for the RODEO Project
Source: JMIR Res Protoc. 2018 Aug 21;7(8):e10473. doi: 10.2196/10473 (PMC6123537; doi:10.2196/10473)
Supplement: Multimedia Appendix 3 [file resprot_v7i8e10473_app3.pdf]

Questionnaire "Willingness to change"

As you know, carrying out the RODEO project means changing work habits. The success of the project is partly determined by employees' willingness to change. To acquire insight in which factors determine the willingness to change we ask you to fill in this questionnaire. We also ask you for possible barriers and facilitators for the change process. We kindly ask you to return the filled out questionnaire to us before **Thursday September 22th 2016**.

**Hospital:**	**Fully agree**	**Somewhat agree**	**Neither agree nor disagree**	**Somewhat disagree**	**Disagree**	**Comments**
WORK RELATED CONSEQUENCES						
1. Reducing unnecessary diagnostics will increase the quality of care						
EMOTIONS						
2. Senior physicians and junior physicians find reducing unnecessary diagnostics challenging.						
3. Senior physicians and junior physicians find reducing unnecessary diagnostics risky.						
EMOTIONAL INVOLVEMENT						
4. Senior physicians and junior physicians experience involvement with the RODEO project.						
ATTITUDES						
5. The Board of Directors fully supports the RODEO project.<br>6. Senior physicians fully support the RODEO project.						
PREVIOUS EXPERIENCE WITH CHANGE						
7. Senior physicians are able to contribute to the success of the RODEO project with their knowledge on the subjects.						
8. Senior physicians have had successful experiences with similar projects.						
TIME						
9. Senior physicians and junior physicians have sufficient time to pay attention to reducing unnecessary diagnostics.						
MANAGEMENT OF THE CHANGE PROCESS						
10. The RODEO project has a clear underlying phasing.						
COMPLEXITY OF THE CHANGE PROCESS						
11. The RODEO project can be introduced with the current means and manpower.						
TEAMS' WILLINGNESS TO CHANGE						
12. In your opinion, are senior physicians and junior physicians willing to commit to the process of change?						
13. In your opinion, are senior physicians and junior physicians willing to overcome possible resistance against the process of change?						
BARRIERS AND FACILITATORS FOR CHANGE						
14. Please name the three, in your opinion most important facilitators of the change process.						

- ...<br><br>- ...<br><br>- ...
15. Please name the three, in your opinion most important barriers of the change process.<br>- ...<br><br>- ...<br><br>- ...
